# The Big Breakfast Study: Chrono‐nutrition influence on energy expenditure and bodyweight

**DOI:** 10.1111/nbu.12323

**Published:** 2018-05-08

**Authors:** L. C. Ruddick‐Collins, J. D. Johnston, P. J. Morgan, A. M. Johnstone

**Affiliations:** ^1^ The Rowett Institute University of Aberdeen Aberdeen UK; ^2^ Faculty of Health and Medical Sciences University of Surrey Guildford UK

**Keywords:** breakfast, chrono‐nutrition, circadian rhythms, energy balance, meal timing, weight loss

## Abstract

A growing body of evidence highlights the importance of the biological clock as a modulator of energy balance and metabolism. Recent studies in humans have shown that ingested calories are apparently utilised more efficiently in the morning than in the evening and this is manifest through improved weight loss, even under iso‐energetic calorie intake. The mechanisms behind this enhanced morning energy metabolism are not yet clear, although it may result from behavioural adaptations or circadian driven variations in physiology and energy metabolism. A major objective of the newly funded *Big Breakfast Study* therefore is to investigate the mechanistic basis of this amplified morning thermogenesis leading to enhanced weight loss, by exploring behavioural and physiological adaptations in energy expenditure alongside the underlying circadian biology. This report briefly discusses the current research linking meal timing, circadian rhythms and metabolism; highlights the research gaps; and provides an overview of the studies being undertaken as part of the Medical Research Council‐funded *Big Breakfast Study*.

## Introduction

The UK has been reported to have one of the highest evening energy intakes, with the proportion of daily energy intake increasing gradually across the day and dinner providing on average 40% of daily calories (Almoosawi *et al*. 2016). Similar patterns of increasing energy intake over the day, albeit with slightly smaller dinner intakes of approximately 33%–34%, have been reported across a number of other nations, including the US, Canada, Germany, Denmark, The Netherlands and Belgium (Almoosawi *et al*. 2016). To date, surprisingly little attention has been paid to the importance of time of day on the nutritional response or energy balance.

**Figure 1:**
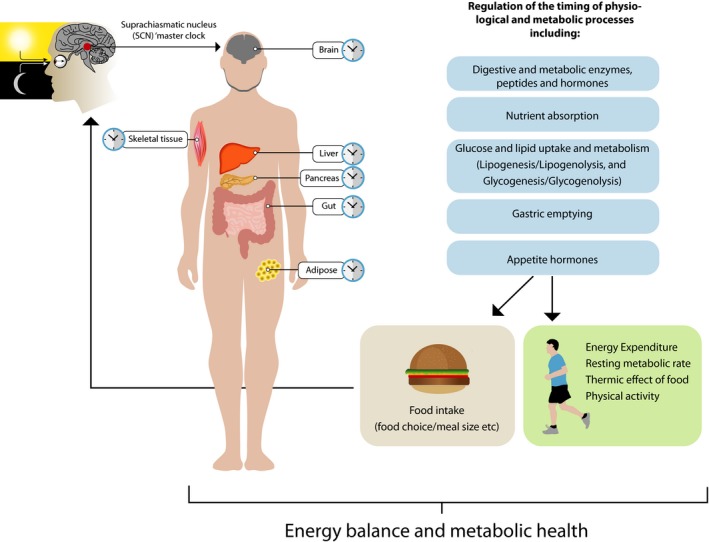
The role of circadian rhythms in regulating metabolic processes and energy balance. Clock genes in peripheral tissues are primarily regulated by the central ‘master clock’ in the hypothalamus (the suprachiasmatic nucleus; SCN), which is predominantly under control by the light/dark cycle. Clock genes are also entrainable by other external factors including food intake and exercise. Clock genes have been established in the brain, liver, gastrointestinal tract, endocrine system adipose tissue and skeletal muscle. They regulate the timing of physiological processes, specifically those involved in the digestion of food, nutrient uptake and nutrient metabolism. These in turn are likely to affect energy expenditure through regulating resting energy expenditure, thermic effect of food and physical activity. Behavioural implications include an influence on food intake and food choice as well as exercise. [Colour figure can be viewed at http://wileyonlinelibrary.com]

The recent Nobel Prize awarded to Jeffrey Hall, Michael Rosbash and Michael Young for their discoveries of the molecular basis of biological rhythms highlights the profound importance of circadian rhythms in biology (chronobiology). Over the last 15 years, there has been increased recognition of how chronobiology impacts on health and disease. Chrono‐nutrition extends chronobiology research and defines the emerging discipline of science surrounding the complex interactions between circadian biology, nutrition and metabolism. Clock genes are now recognised for their indisputable role in coordinating nearly all biological and physiological processes of the body. The suprachiasmatic nucleus (SCN) of the hypothalamus, the ‘master clock’, is primarily regulated by light/dark cycles in order to synchronise the body to the light cycle or solar day (Takahashi 2017). Additionally, peripheral clock genes have been identified throughout the body in nearly all individual tissues and organs, which regulate the timing of physiological functions within these specific tissues. Critically, rhythms persist in tissue/cell culture, indicating that these peripheral clock gene rhythms reflect local clocks. The SCN acts as the master timekeeper, keeping the peripheral clocks in synchrony to the central 24‐hour circadian rhythm. Nonetheless, other external factors, including food and physical activity, can influence the timing of peripheral clocks (Bass 2012; Jiang & Turek 2017). Circadian rhythmicity influences the essential processes of metabolism and energy expenditure, with rhythmic expression of clock genes identified in tissues of the gut, liver, endocrine organs, adipose tissue and skeletal muscle. These in turn control the timing of digestion, nutrient uptake and metabolism, hormonal and metabolite regulation, appetite, ingestive behaviour and physical activity (Bass 2012; Jiang & Turek 2017) (Fig. 1). The relationship between circadian rhythms and metabolism involves a complex feedforward and feedback system. In addition to postprandial responses being influenced by circadian rhythms, food intake itself can entrain circadian clocks in tissues such as the liver, intestines and adipose tissue (Damiola *et al*. 2000; Hara *et al*. 2001; Froy *et al*. 2005; Wehrens *et al*. 2017), modulating both gene expression and physiological functions. Despite the growing knowledge of the interactions between nutrition and circadian biology, much remains to be understood about how meal timing may impact on health and predominantly nutritional‐based diseases such as obesity, type 2 diabetes and cardio‐metabolic disorders. The current literature is dominated by pre‐clinical studies, and there is strong need for more translational studies in humans. The proposed work addresses this gap, providing a novel and timely project, which draws upon the existing literature in animal models, yet sets up mechanistic, hypothesis driven intervention studies in human models.

## Timing of eating (calorie distribution): Effects on energy balance and metabolic health

**Figure 2:**
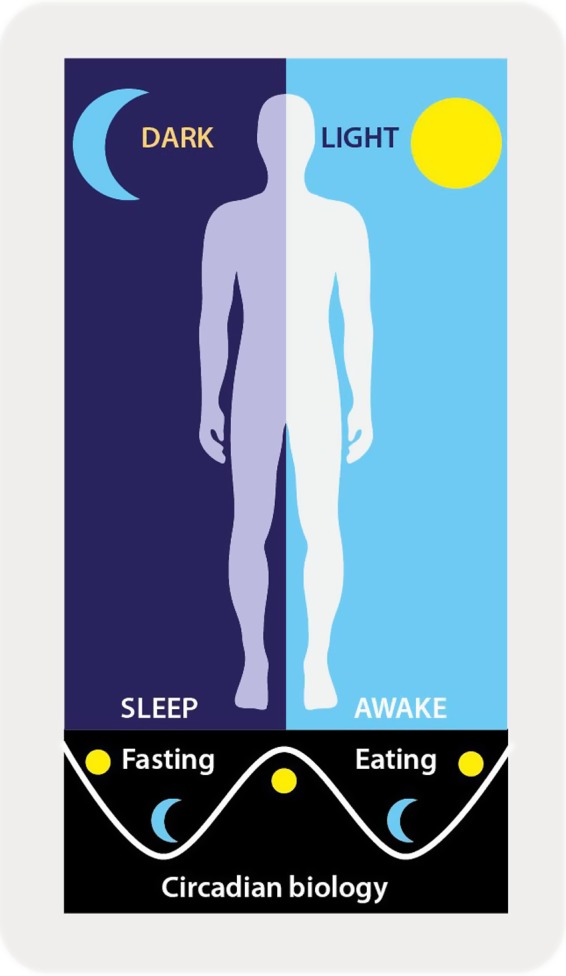
Representation of synchrony between the light/dark, wake/sleep and feed/fast cycles in humans as a diurnal species. [Colour figure can be viewed at http://wileyonlinelibrary.com]

Dietary advice for weight management in humans is based on the assumption that ‘a calorie is a calorie’ and that meal timing is inconsequential. However, recent evidence from chrono‐nutrition studies questions this assumption and verifies the importance of meal timing in energy balance and cardio‐metabolic health and disease (Antunes *et al*. 2010; Garaulet *et al*. 2013; Jakubowicz *et al*. 2013, 2017). Humans are a diurnal species with the light cycle stimulating wakefulness and feeding, and darkness initiating sleeping and fasting (Fig. 2). Misalignment between normal feed/fast, day/night and sleep/wake cycles may desynchronise central and peripheral regulations of metabolic processes and contribute to obesity and metabolic disorders (Scheer *et al*. 2009; Antunes *et al*. 2010). Increased risk of obesity and related health conditions has been associated with breakfast skipping and late night feeding (Ma *et al*. 2003; Cleator *et al*. 2012; Fong *et al*. 2017), indicating morning energy intake may have substantial health benefits. A recent meta‐analysis of observational studies demonstrated an association between evening energy consumption and higher BMI (Fong *et al*. 2017) and McHill *et al*. (2017) found that, on average, obese individuals consumed most of their calories an hour closer to melatonin onset (biological marker of impending sleep onset) compared to lean individuals. In addition to significantly increasing the risk of obesity, a recent study observed that acute breakfast omission altered clock genes expression and resulted in an increased postprandial glycaemic response (Jakubowicz *et al*. 2017). Additionally, shift workers are predisposed to weight gain and metabolic disorders, which has been suggested to arise from circadian misalignment (Antunes *et al*. 2010). Intentionally inducing misalignment in sleep/wake and feed/fast times in mice models through feeding during the light phase (normal fasting period) resulted in significantly greater weight gain compared to mice fed during the dark phase. This was despite equivalent calorie consumption and locomotion, demonstrating the capacity of meal timing to modify energy balance and bodyweight (Arble *et al*. 2009).

Preliminary dietary intervention studies in humans seem to suggest that calories ingested at different times of the day have different effects on energy utilisation, leading to differential weight loss, even at iso‐caloric amounts (Garaulet *et al*. 2013; Jakubowicz *et al*. 2013). Garaulet *et al*. (2013) found that late lunch eaters lost significantly less weight (2.1 kg) than early lunch eaters over a 20‐week protocol. This was despite no reported differences in energy intake, dietary composition, estimated energy expenditure, appetite hormones or sleep duration. Similarly, Jakubowicz *et al*. (2013) reported that during a 12‐week sub‐maintenance diet in 93 overweight women, morning calorie consumption (50% calories at breakfast and 14% at dinner) resulted in a 5.1 kg greater weight loss relative to evening calorie consumption (14% calories at breakfast and 50% at dinner), with weight loss differences reaching statistical significance by week 4. Morning calorie consumption was also associated with greater improvements in fasting glucose, insulin and triglycerides, glucose tolerance as well as lower hunger scores. The mechanisms involved in this mealtime‐dependent differential weight loss are unclear, but they may include the following: (1) behavioural adaption such as altered (increased or decreased) physical activity or energy expenditure at other times of the day, and/or (2) the influence of normal biological circadian/diurnal rhythms on energy metabolism at different times of the day. Significantly greater satiety was an additional outcome of morning compared to evening‐loaded calorie consumption (Jakubowicz *et al*. 2013). While these studies point to new and interesting roles of meal timing in energy balance, the effects observed may simply have been due to higher energy intake and/or non‐compliance in the later lunch and evening predominant feeding patterns, which was unaccounted for with their methods of assessing dietary intake.

Despite this evidence, clinical trials comparing meal timing in humans are extremely limited. To date, no randomised, controlled crossover studies comparing large breakfast meals vs. large evening meals have been conducted. The *Big Breakfast Study* will address this gap in research by implementing a randomised control trial comparing morning‐loaded vs. evening‐loaded weight loss diets in overweight and obese individuals, in which all components of energy intake and energy expenditure will be monitored over 4‐week periods.

## Physiological mechanisms and circadian influence on energy metabolism

Rhythmic gene expression has been identified in nearly all tissues and organs of the body. In addition, many of these rhythmic genes have identified roles in the regulation of metabolic processes and energy expenditure, such as digestion, nutrient absorption, lipid and glucose metabolism and locomotion (Bass 2012; Sahar & Sassone‐Corsi 2012; Jiang & Turek 2017) (Fig. 1). Importantly, a large number of these genes are involved in the rate‐limiting steps of metabolic pathways (Sahar & Sassone‐Corsi 2012) providing a clear theoretical basis for a chronobiological influence on metabolism. A number of postprandial metabolic processes show time of day variations, with faster gastric emptying, enhanced intestinal absorption, superior glucose tolerance and higher postprandial energy expenditure [Thermic Effect of Food (TEF): the increase in energy expenditure after meal consumption associated with the energy costs of nutrient digestion and storage] observed in the morning compared to the evening. A number of studies have shown a strong time of day effect of TEF; specifically it has been demonstrated to be significantly higher in the morning relative to the evening (Romon *et al*. 1993; Bo *et al*. 2015; Morris *et al*. 2015). However, diurnal differences in the TEF are not consistently found in all studies (Weststrate *et al*. 1989). The TEF consists of both obligatory (essential energy expenditure for digestion, nutrient absorption and nutrient storage) and facultative components (energy expenditure associated with increased postprandial sympathetic nervous system activity) (Ravussin *et al*. 1985). Thus, the higher TEF in the morning, found in a number of studies, may be a result of circadian influences associated with either higher costs of essential energy requirements for digestion or increased sympathetic nervous system activity, or simply a result of meal size. The TEF response also appears to be under endogenous circadian control, as shifts in day/night and sleep/wake times alters the timing of TEF accordingly. This is evident through the use of phase shift protocols, where the light/dark cycle is either abruptly advanced or delayed, which temporarily desynchronises an individual's internal body clocks from the ambient light/dark cycle. The effects of this desynchrony can then be seen in terms of the timing of sleep/wake and feed/fast cycles. This mimics what an individual would experience on a transatlantic flight or is regularly experienced by shift workers following night shift work. Morris *et al*. (2015) studied the effect of a 12‐hour phase delay on TEF in human subjects. They showed that normally TEF is higher in the morning compared to the evening. This was maintained 1 day after the phase delay, but after 3 days, this difference was lost. It is important to note that the time points for all TEF measurements were locked onto the initial light/dark cycle and were not changed to follow the phase delay. The TEF measurements then reflected the status of the body's internal biological clock (and rhythms). The loss of the clear morning/evening difference by day 3 reflects the gradual resynchronisation to the new light/dark cycle and readjustment to establish a greater morning TEF. It is also important to note that TEF in this study was only measured for 2 hours, where in fact the TEF response may last as long as 6 hours (Reed & Hill 1996). Therefore, differences in the timing of the morning vs. evening TEF response, as a result of changes in the rate of gastric emptying and nutrient absorption, may have been missed and have influenced the results. Further research which measures TEF over longer durations to capture the entire response is necessary to address diurnal variations in the TEF and circadian influences.

Diurnal variations are evident in gastrointestinal absorption rate and expression of gastrointestinal genes involved in nutrient transport, which are both typically higher during the active phase and following fasting (Sotak *et al*. 2011; Hussain & Pan 2015). Rates of gastric emptying have also been shown to be under circadian control, with slower emptying of the stomach in the biological evening (Goo *et al*. 1987). These factors may contribute to the observed elevated morning TEF, by influencing the release of nutrients into the intestines for absorption, and also affecting the release of hormones and metabolites required for energy consuming processes of nutrient digestion and storage (Weststrate *et al*. 1989; Romon *et al*. 1993). It is not yet clear whether the day/night rhythm in gastrointestinal absorption rate is driven by the behavioural cycle or endogenous circadian biology, and no studies have considered how 24‐hour variations in gastric emptying following a meal may differentially influence postprandial energy metabolism across a day. Reduced evening insulin release and glucose tolerance may further impact on the thermogenic costs associated with glycogenesis following carbohydrate intake and reduce evening TEF (Ravussin *et al*. 1985; Van Cauter *et al*. 1997).

Differential weight loss between morning and evening feeding may also be a result of behavioural influences involving voluntary and incidental physical activity, as well as appetite and food choice. For example, Betts *et al*. (2014) found higher levels of physical activity in breakfast‐consuming individuals relative to those who skipped breakfast. It is also plausible that the difference in energy expenditure between morning and evening meal consumption may reflect altered basal metabolic rate. Thus, in addition to TEF, daily changes in basal metabolic rate and physical activity require careful monitoring in the study of the mechanisms underpinning the relationship between meal timing and bodyweight.

In summary, there is evidence that timing of eating has a clinically meaningful influence on weight loss, energy balance and metabolic health. However, the underlying mechanisms contributing to meal‐time‐induced differential energy expenditure are not yet clear and the variable components of daily energy expenditure have never been explicitly studied in terms of daily rhythms.

## Objectives of the Big Breakfast Study

**Figure 3:**
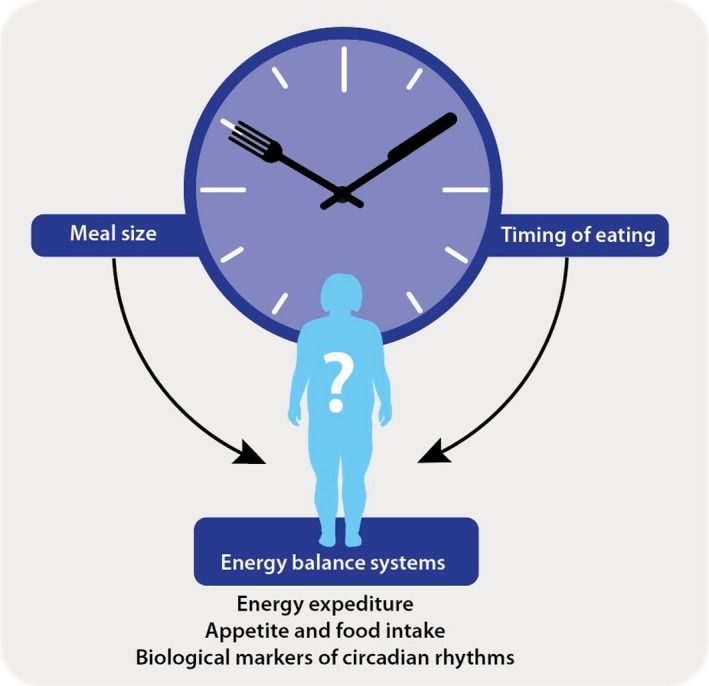
Key objectives of the *Big Breakfast Study* are to understand how altering meal size (calorie intake), specifically morning vs. evening distribution of energy intake, affects energy balance. We will determine the underlying mechanisms of energy balance (through assessing energy expenditure and appetite) and the influence of circadian biology. [Colour figure can be viewed at http://wileyonlinelibrary.com]

Growing evidence suggests some truth to the adage ‘Breakfast like a king and dine like a pauper’, with meal timing appearing to influence energy balance and bodyweight (and thus, disease risk). The *Big Breakfast Study* is funded by the Medical Research Council to investigate the underlying biological and/or behavioural drivers that influence energy balance, and thus bodyweight in overweight and obese persons, relative to daily energy distribution. The key objectives of the project are schematically represented in Figure 3. The project will investigate how the components of energy expenditure (*i.e*. total daily energy expenditure, resting metabolic rate, TEF, physical activity) are influenced by meal timing, circadian and behavioural effects. The aim is to understand the underlying mechanisms (*i.e*. hormones/metabolites/gastric emptying), that determine time of day differences in energy expenditure and thereby make consuming the largest meal of the day at breakfast (and smaller evening meal), important in influencing daily energy balance. The project will combine specialised techniques (*i.e*. use of stable isotopes for measuring total daily energy expenditure, gastric emptying and total body water; and circadian control, through the use of controlled light and dark exposure, and sleep and meal times) and protocols with complete dietary control to assess the physiological and behavioural factors influencing the relationship between external clock time, internal circadian rhythms, energy expenditure and energy balance (body mass).

Two studies will explore potential behavioural and circadian/diurnal influences on energy expenditure and energy balance. Specifically, the project will address two overriding objectives:

1. to assess the impact of timing of eating on mechanisms associated with energy expenditure and substrate utilisation;
2. to test the contribution of biological circadian influence on energy expenditure and related endocrine pathways during a fixed feeding phase shift protocol.

**Figure 4:**
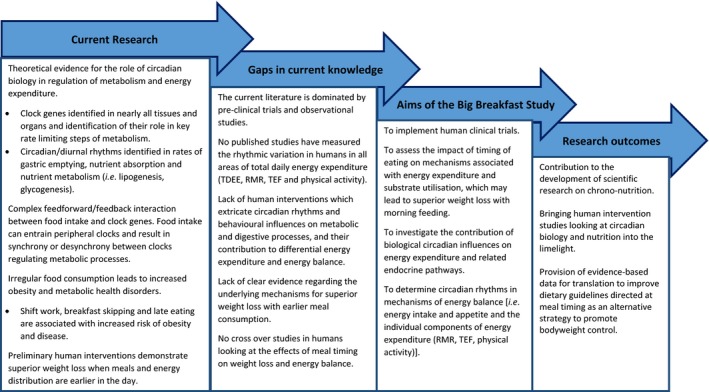
Summary of current research, current gaps in the literature and the aims of the *Big Breakfast Study*. TDEE, total daily energy expenditure; RMR, resting metabolic rate; TEF, thermic effect of food. [Colour figure can be viewed at http://wileyonlinelibrary.com]

These studies will extend current knowledge about the interaction of diet and the body's circadian rhythms (‘chrono‐nutrition’) and help to inform future development of nutritional guidelines for weight control, based on optimising the timing of energy consumption. An overview of the current literature, research gaps and aims of the current project are reviewed in Figure 4.

### Objective 1: To assess the impact of timing of eating on mechanisms associated with energy expenditure and substrate utilisation: The Mealtime Study

The *Mealtime Study* aims to answer the questions: ‘Does morning‐loaded energy consumption significantly influence energy balance and therefore weight loss compared to evening‐loaded energy consumption?’ and ‘What are the underlying mechanisms leading to differential weight loss due to meal timing?’ The study will implement a 10‐week randomised, crossover energy restrictive dietary intervention study designed for weight loss in overweight or obese, but otherwise healthy, adults comparing morning‐ and evening‐loaded feeding. The dietary intervention will involve two 4‐week weight loss phases with calories distributed predominantly in the morning (45%, 35%, 20% of energy intake at breakfast, lunch and dinner, respectively) or the evening (20%, 35%, 45% of energy intake at breakfast, lunch and dinner, respectively) with 1‐week weight maintenance diets at baseline and mid‐study between the two weight loss phases (washout). All diets will be prepared at the Rowett Institute for participants to collect and consume outside the Institute. The primary outcome measure, bodyweight, will be measured three times per week throughout the 10‐week protocol, when participants attend the Institute's human nutrition unit for measurement and food collection. All other outcome measures will be assessed at the end of each study phase (weight maintenance 1, energy restriction diet 1, weight maintenance 2 and energy restriction diet 2).

Changes in body composition will be assessed using the four compartment model (bone mineral, water, fat mass and fat‐free mass). Doubly‐labelled water, which remains the gold standard for assessing total energy expenditure (Buchowski 2014), will be used for the 8 weeks of energy restriction to objectively measure total energy expenditure. This will provide a precise assessment of whether meal timing affects total daily energy expenditure during energy restriction. We will also assess all components of energy expenditure. This will include the use of actigraphs to assess physical activity as well as indirect calorimetry to measure resting metabolic rate (RMR) as well as TEF following breakfast meals [calorically matched to the corresponding study phase (*i.e*. weight maintenance 1 or 2, big breakfast or small breakfast energy restriction diet)]. This will enable us to determine whether differences in energy expenditure and energy balance in relation to timing of energy intake are due to behavioural changes (*i.e*. variations in incidental physical activity) or changes in physiological energy expenditure (RMR and TEF). Breakfast skipping has been correlated to lower levels of physical activity (Aarnio *et al*. 2002; Sandercock *et al*. 2010; Betts *et al*. 2014) and increased sedentary leisure time (Amigo‐Vázquez *et al*. 2016), and it is therefore possible that morning feeding will promote activity. Although participants will be instructed not to alter their physical activity throughout the study, incidental and subconscious increases in activity may occur following morning feeding and result in increased non‐resting energy expenditure and greater energy deficit.

It has been proposed that the lower TEF in the evening found in previous studies may be as a result of slower gastric emptying or reduced nutrient uptake. We will therefore measure gastric emptying by stable isotope technique (^13^C Octanoic Acid) at the same time as assessing TEF and postprandial changes in glucose, insulin and gut hormones. While previous studies have indicated a greater morning TEF (Morris *et al*. 2015), the use of just 2 hours of measures is insufficient to determine the entire TEF response. Six‐hour measures of TEF and gastric emptying will be applied based on the methodology by Reed and Hill (1996), which is a more discriminating way to measure the entire TEF response.

In line with previous research, we will also assess measures of metabolic health on test days at the end of each study phase. This will include blood pressure and fasting and postprandial glucose, insulin and lipids to determine other health benefits associated with morning‐loaded feeding. Blood glucose will be further assessed over the final 3 days of each study phase using continuous glucose monitors (CGMs). It has previously been shown that morning feeding resulted in significantly greater reductions in fasting glucose, insulin, homeostatic model assessment‐insulin resistance and serum triglyceride, and significantly greater improvements in high‐density lipoprotein (HDL)‐cholesterol, compared to evening feeding (Jakubowicz *et al*. 2013). The *Mealtime Study* will help to solidify this evidence by implementing a crossover design for within‐individual comparisons of metabolic blood substrates. The inclusion of CGMs will allow detailed assessment of the impact of morning‐loaded feeding on acute postprandial glucose responses and also daily glucose fluctuations.

Lastly this study will assess the participants’ subjective appetite using Visual Analogue Scales (Flint *et al*. 2000) measured hourly for three consecutive days during the last week of each study phase, during free living conditions and concurrent with continuous glucose assessment and actigraph assessment. Appetite ratings will also be taken every 30 minutes for the entire test day simultaneous to the measures of gastric emptying and TEF. From this we aim to determine how meal timing affects daily appetite, as well as establish complex relationships between blood glucose, physical activity, gastric emptying and energy expenditure.

### Objective 2: To test the contribution of biological circadian influence on energy expenditure and related endocrine pathways during a fixed feeding phase shift protocol: Effects of a 5‐hour phase delay of light and behaviour on daily rhythms of human metabolism

Separating human environmental and behaviour influences from endogenous circadian physiology will assist in understanding the underlying mechanisms associated with the rhythmic expression of energy expenditure. Specifically, the use of phase shift protocols, which alter an individual's relative time of day to earlier or later, by advancing or delaying the light/dark, sleep/wake and feed/fast cycles, can be used to ascertain the relative contribution of endogenous circadian rhythms vs. behavioural cycles. This study aimed to answer the question ‘How are the patterns of energy expenditure, metabolism and gastric emptying and related endocrine pathways affected by circadian rhythms during a fixed feeding phase shift protocol and how do endogenous circadian rhythms influence energy expenditure and energy balance?’

Participants will attend the research facility for a 7‐day in‐house stay, including 2 baseline days and 5 days following an acute 5‐hour phase shift. The 5‐hour phase shift will involve a 5‐hour delay in timing of light and dark exposure, sleep and wake times and all meal times so that during baseline, sleep will be permitted from 23:00 to 07:00, while following the phase shift, sleep will be permitted from 04:00 to 12:00. Breakfast will be 1 hour after waking, lunch 5 hours later and dinner 5 hours after lunch. This will permit the retaining of an 8‐hour sleep opportunity and thereby minimising any possible effect of acute sleep deprivation. Outcome measures will be assessed on day 2, prior to the phase delay, and on days 3, 5 and 7 (days 1, 3 and 5 after the phase delay), which will allow us to investigate whether metabolic rhythms realign to the new behavioural and light/dark rhythms over the 5 days following the phase delay. The study design allows postprandial responses to be assessed at the same clock time immediately before and after the phase shift (*i.e*. breakfast and lunch on day 3 occur at exactly the same clock time as lunch and dinner on day 2). Daily energy intake for participants will be set at 1.1 times RMR, which is used as the energy intake required to meet weight maintenance requirements under test conditions. Energy intake will be consumed as three iso‐caloric meals at breakfast, lunch and dinner (20% protein, 35% fat and 45% carbohydrate). By analysing participants over the 5 days following the phase shift, we will be able to follow the realignment of each participant's internal rhythms of energy expenditure, metabolism and related endocrine pathways with the newly imposed light/dark cycle.

Light and dark exposure (both time and lux) will be controlled on all days with participants blinded to time of day so as not to interfere with their natural circadian rhythm. The gold standard marker of human circadian phase, plasma melatonin concentration, will be measured every hour for up to 15 consecutive samples (*e.g*. between 16:00 and 06:00). Data will be analysed by calculating the timing of melatonin signal onset, according to validated methodologies (Benloucif *et al*. 2008). Continuous glucose monitoring and measurements of physical activity using actigraphs will also be undertaken as per the *Mealtime Study* and worn for the entire 7‐day protocol.

Resting metabolic rate will be assessed on the morning of all test days and TEF assessed over 5 hours following all three meals of the day: breakfast, lunch and dinner. This will enable us to quantify TEF to specific meals, as well as total TEF across an entire day, and determine changes in response to the phase shift. By simultaneously measuring gastric emptying and metabolites (glucose, insulin, gut hormones and lipids) at the breakfast meals, we will be able to identify whether there are differences in the rate or magnitude of the TEF response in relation to the phase shift and whether these are attributable to differences in gastric emptying, substrate uptake and utilisation. This phase shift study will enable us to assess the influence of the endogenous circadian clock in the regulation of these digestive and metabolic processes and on the individual components of energy expenditure (RMR, TEF, physical activity).

## Beneficiaries and public health message

Our current work aimed to investigate whether the typical meal consumption pattern in the UK, of having a small breakfast and large evening meal, is working against internal biological rhythms and exacerbating difficulties in successful weight management. Complementary research supported by the Scottish government's Rural and Environmental Science and Analytical Services is currently being undertaken at the Rowett Institute to examine how breakfast meal composition (45% calories consumed at breakfast, comparing high fibre vs. high protein) may also affect energy balance and assist in weight management in a similar 10‐week weight loss crossover study. Currently, there are no dietary guidelines specific to meal timing and particularly, no guidelines in relation to shift workers. Together these studies will provide evidence‐based data for translation to improve dietary guidelines directed at meal timing as an alternative strategy to promote bodyweight control.

## Dissemination

Findings from the *Big Breakfast Study* will be disseminated to an extensive audience including the scientific community, industry, public and independent bodies and the general public. Specifically, we will produce infographics, short video summaries, press release statements, and written guidelines and media for presentation and distribution to industries (*e.g*. Scottish Food and Drink Federation), health bodies (*e.g*. NHS) and independent bodies, as well as presenting information at local and UK‐wide public events (*e.g*. Aberdeen's Café Med and Food Matters Live). These broadcasts will be used to highlight the study findings, educate important stakeholders and provide evidence for development of health policy and support opportunities for future studies.

## Conclusions

Early evidence supports morning‐loaded energy distribution as a beneficial strategy for weight control. The *Big Breakfast Study* aims to explore the mechanistic basis for such findings and determine the contribution of the endogenous circadian system to energy expenditure and energy balance. This under‐researched area of nutritional science may lead to innovative knowledge, which could pave new ways of addressing metabolic health and obesity via implementing dietary guidelines directed at meal timing.

## Funding

The study is funded by the Medical Research Council.

## Author contributions

Alex Johnstone, Leonie Ruddick‐Collins, Peter Morgan and Jonathon Johnston all contributed to the writing and preparation of the manuscript.

## Conflict of interest

JDJ has performed consultancy work for Kellogg Marketing and Sales Company (UK) Limited and has a research collaboration with Nestlé Research Centre. No conflict of interest has been declared by any other author.
